# Context Matters: Response Heterogeneity to Collagen-Targeting Approaches in Desmoplastic Cancers

**DOI:** 10.3390/cancers14133132

**Published:** 2022-06-26

**Authors:** Ashley M. Fuller, Tzipora S. Karin Eisinger-Mathason

**Affiliations:** Abramson Family Cancer Research Institute, Department of Pathology and Laboratory Medicine, Penn Sarcoma Program, Perelman School of Medicine, University of Pennsylvania, Philadelphia, PA 19104, USA; ashley.fuller@pennmedicine.upenn.edu

**Keywords:** desmoplasia, extracellular matrix, tumor microenvironment, stromal depletion, cancer-associated fibroblast, collagen biosynthesis

## Abstract

**Simple Summary:**

A common feature of tumor types such as breast cancer, prostate cancer, pancreatic cancer, and soft-tissue sarcoma is the deposition of collagen-rich tissue called desmoplasia. However, efforts to control tumor growth by disrupting desmoplasia, collectively known as “collagen-targeting approaches”, have had mixed and contradictory results, sometimes even within the same cancer type. We believe that this phenomenon may be due—at least partially—to the fact that “collagen” is not a single molecule, but rather a diverse molecular family composed of 28 unique collagen types. Therefore, in this review, we discuss the diversity of collagen molecules in normal and cancer tissue, and explore how collagen heterogeneity relates to the mixed efficacy of collagen-targeting approaches for cancer therapy.

**Abstract:**

The deposition of collagen-rich desmoplastic tissue is a well-documented feature of the solid tumor microenvironment (TME). However, efforts to target the desmoplastic extracellular matrix (ECM) en masse, or collagen molecules more specifically, have been met with mixed and sometimes paradoxical results. In this review, we posit that these discrepancies are due—at least in part—to the incredible diversity of the collagen superfamily. Specifically, whereas studies of “collagen-targeting” approaches frequently refer to “collagen” as a single molecule or relatively homogeneous molecular family, 28 individual collagens have been identified in mammalian tissues, each with a unique structure, supramolecular assembly pattern, tissue distribution, and/or function. Moreover, some collagen species have been shown to exert both pro- and anti-neoplastic effects in the desmoplastic TME, even within the same cancer type. Therefore, herein, we describe the diversity of the collagen family in normal tissues and highlight the context-specific roles of individual collagen molecules in desmoplastic tumors. We further discuss how this heterogeneity relates to the variable efficacy of “collagen-targeting” strategies in this setting and provide guidance for future directions in the field.

## 1. Introduction

Collagens are the most abundant proteins in mammals, comprising approximately 30% of an individual’s total protein mass, and are distributed ubiquitously throughout the extracellular matrices (ECMs) of mammalian tissues [1]. Twenty-eight individual collagen species have been documented to-date, each of which exhibits a unique structure, tissue expression pattern, and/or function. Under physiologic conditions, collagen molecules play fundamental roles in maintaining tissue organization/architecture, and regulate critical aspects of the wound-healing response such as cell differentiation, proliferation, adhesion, and migration [1]. In cancer, however, chronic or aberrant activation of these processes can lead to the development of desmoplasia, the deposition of a fibrotic, collagen-rich ECM within the tumor microenvironment (TME), with implications for tumor initiation, progression, therapeutic responses, and patient prognosis [2]. Accordingly, the development of “collagen-targeting” or “stromal depletion” strategies for cancer therapy is an area of significant research interest. However, these approaches have had diverse, and in some cases, paradoxical, effects on tumor progression, demonstrating that the functions of collagen molecules in the desmoplastic TME are highly complex and likely context-specific.

We hypothesize that one possible explanation for these observed discrepancies is that, despite the well-documented diversity of the collagen superfamily, many studies of “collagen-targeting” approaches have considered “collagen” to be a single molecule or a relatively homogeneous molecular family. Moreover, few studies have characterized the effects of these interventions on the structure, expression, deposition, etc. of individual collagen species, or on the desmoplastic tumor ECM en masse, and how these alterations impact other aspects of tumor biology. Therefore, in this review, we posit that a systematic investigation of the functions of individual collagen molecules in tumor initiation and progression, together with a more comprehensive understanding of how these proteins are impacted by targeted therapies, is critical. To this end, we first summarize the roles of individual collagen molecules in both physiologic and malignant settings, focusing particularly on specific molecular species for which both pro- and anti-neoplastic functions have been documented. We then discuss the efficacy of select “collagen-targeting” strategies in desmoplastic cancers, highlighting approaches that have exhibited contradictory results depending on tumor context.

## 2. Collagens in Physiologic Tissue Contexts

### 2.1. The Collagen Family: Nomenclature, Structure, and Function

In vertebrates, the collagen superfamily consists of 28 unique members numbered with Roman numerals in the order of their discovery. All collagens are composed of three homotrimeric or heterotrimeric polypeptide chains (α chains) that are designated with Arabic numerals followed by the collagen type in parentheses (e.g., “α1(I)” denotes the α1 chain of collagen I). Similarly, the genes encoding collagen α chains begin with the prefix “COL”, followed by the Arabic numeral for the collagen type, the letter “A” for α chain, and the Arabic numeral for the specific collagen chain (e.g., *COL1A1* encodes the α1 chain of collagen I, or the α1(I) chain).

Collagens exhibit a diverse array of structural properties and can be divided into multiple sub-families according to their structure or supramolecular assembly pattern (Table 1). The fundamental structural properties of each subfamily, as well as the members and functions thereof, are described in brief below.

#### 2.1.1. Fibrillar Collagens (Collagens I, II, III, V, XI, XXIV, and XXVII)

A structural motif common to all collagen α chains is the presence of at least one triple-helical (collagenous) domain characterized by repeating glycine-X-Y sequences. These sequences frequently contain proline and hydroxyproline in the X and Y positions, respectively, which impart stability to the mature collagen molecule [37]. The three constituent α chains of a given collagen molecule are then coiled about a common axis to form a triple-helix. The presence of glycine, which possesses a hydrogen atom side chain, in every third position of the collagenous domain facilitates the tight packing of this superhelical structure [37]. A singular long, central, triple-helical domain is the predominant feature of fibrillar collagen α chains [1]; thus, most fibrillar collagen molecules resemble the classical quarter-staggered rope-like fibrils that are visible by electron microscopy [5]. (Refer to Section 4.2.1 for a more detailed discussion of collagen synthesis).

Under physiologic conditions, collagen I is the most prevalent mammalian collagen, comprising ~90% of the body’s collagen content. Collagen I is expressed in virtually all connective tissues, including bone, and is a major structural protein in skin, the eyes, and the vasculature [3]. Like collagen I, collagen III is also relatively ubiquitous (5–20% of mammalian collagen content) [6]. This collagen molecule forms a fine meshwork of narrow-diameter reticular fibers that support the cellular components of visceral organs (e.g., liver; lymphoid organs) and loose connective tissue (e.g., adipose) [7]. Collagens I and III are also critical elements of the wound healing response; expression of collagen III predominates in early stages of this process, whereas collagen I is more highly expressed at later stages [4]. In contrast to collagens I and III, the tissue distributions of collagens II, V, and XI are more restricted. Collagens II and XI are quantitatively major and minor components, respectively, of cartilage, whereas collagen V is a quantitatively minor constituent of the cornea, often found in association with collagen I [5]. Finally, relatively little is known about the most recently discovered fibrillar collagens, collagens XXIV and XXVII. However, both molecules appear to play important roles in various aspects of skeletogenesis including osteoblast differentiation [29,30] and endochondral ossification [32,33].

#### 2.1.2. Non-Fibrillar Collagens

The remaining collagen species are broadly classified as non-fibrillar collagens, the majority of which can be further divided into one of four subfamilies. The largest subfamily, fibrillar-associated collagens with interrupted triple-helices (FACITs), consists of collagens IX, XII, XIV, XVI, XIX, XX, XXI, and XXII. As the name implies, FACITs contain multiple short triple-helical domains that are interrupted by non-collagenous (i.e., non-triple-helical) domains, and are often found in association with fibrillar collagen molecules. For example, collagens XII and XIV are associated with collagen I fibrils [17], whereas collagens IX and XVI are found in association with collagens II and XI in cartilage [15].

The members of a second non-fibrillar collagen subfamily, the multiplexins (collagens XV and XVIII), are similar to the FACITs in that they also contain multiple interrupted triple-helical domains. These collagen species are predominantly localized to the basal laminae of various tissues [21,22]. Interestingly, proteolytic cleavage of the C-terminal domain of both collagen XV and XVIII results in the production of biologically active fragments (matrikines/matricryptins; restin and endostatin, respectively) that possess anti-angiogenic properties [21].

The network-forming collagens (collagens IV, VIII, and X) constitute the third non-fibrillar collagen family. Collagens VIII and X form hexagonal molecular networks in highly specialized tissues such as Descemet’s membrane of the eye and hypertrophic cartilage (i.e., in the context of endochondral ossification), respectively [5,13,14,16]. In contrast, collagen IV exhibits a more ubiquitous tissue distribution, forming networks of complex polygonal lattices in all basal laminae of the body [8,9,10].

Transmembrane collagens (collagens XIII, XVII, XXIII, and XXV) constitute the final non-fibrillar collagen subfamily. These intriguing species exist as both full-length transmembrane proteins and soluble ectodomains [18,19]. The most thoroughly studied transmembrane collagen, collagen XVII, is found in skin hemidesmosomes where it plays a crucial role in anchoring epidermal cells to the underlying dermis [18,19]. Collagens XIII and XXIII are similarly found in cutaneous tissue [19]; however, recent work has also reported the expression of various transmembrane collagen species in select neuronal subsets [28] and neuromuscular junctions [20].

The remaining non-fibrillar collagens, collagens VI, VII, XXVI, and XXVIII, have not been assigned to any of the aforementioned subfamilies. The most well studied of these species, collagen VI, forms a network of linked “beaded filaments” with the non-collagenous domains representing the “beads” [1]. This collagen is found ubiquitously in mammalian tissues, but plays a particularly important role in supporting the cell membranes of skeletal muscle myocytes [11]. In contrast, collagens VII, XXVI, and XXVIII display a more limited expression distribution, and are localized to anchoring filaments [12], gonadal tissue [31], and non-myelinated regions of the peripheral nervous system [36], respectively.

## 3. Context Matters: Individual Collagens Can Have Both Pro- and Anti-Neoplastic Roles in the TME

### 3.1. Pathologic Collagen Dynamics: Desmoplasia in the TME

#### 3.1.1. Origins of Tumor Desmoplasia

Due to the prevalence and diversity of collagen species in normal mammalian tissues, it is unsurprising that dysregulations in collagen-associated processes (e.g., expression, synthesis, post-translational modification, deposition, degradation, and remodeling) are hallmarks of numerous pathologies [40,41,42]. In the context of cancer, dysregulated collagen homeostasis manifests as desmoplasia, the aberrant deposition of a dense, collagen-rich ECM within the tumor proper [42,43]. Initially thought to reflect the condensation of pre-existing collagen fibers, desmoplasia is now understood to result from the increased de novo synthesis of collagens and other ECM proteins [44,45,46]. Desmoplasia is particularly prevalent in breast, prostate, and pancreatic carcinomas (pancreatic ductal adenocarcinoma; PDAC) [2,47,48], as well as in soft-tissue sarcomas (STS), a heterogeneous group of mesenchymal tumors derived from connective tissues such as muscle, adipose, and fibrous connective tissue [49]. Desmoplasia can further be observed in non-malignant tissues such as radiographically dense normal breast, where it is strongly associated with increased breast cancer risk [50,51]. Thus, desmoplasia can be both a response to the presence of invasive cancer cells and a pre-existing factor that fosters the development of a tumor-supportive niche [43].

In STS, the cell types responsible for aberrant ECM deposition have not been conclusively identified, but studies of subtypes such as undifferentiated pleomorphic sarcoma and rhabdomyosarcoma suggest that sarcoma cells themselves may be culpable [49,52]. This phenomenon has been more extensively studied in epithelial tumors, where, unlike in sarcomas, cancer-associated fibroblasts (CAFs) have been implicated as the primary source of matrix proteins in the TME. Accumulating evidence suggests that CAFs in solid tumors can arise from stem cell populations such as mesenchymal and hematopoietic stem cells, as well as from differentiated cell types such as tissue-resident fibroblasts, stellate cells (e.g., in pancreatic and liver carcinomas), adipocytes, endothelial cells, and pericytes (reviewed in [53]). As a result of this heterogeneity, a universal CAF biomarker has been elusive [54]. Moreover, traditional CAF markers such as α-smooth muscle actin (α-SMA), S100A4, fibroblast activation protein (FAP), vimentin, and platelet-derived growth factor receptors (PDGFRα/β) are not exclusively expressed on CAFs and may have context-specific functions [54]. Thus, the precise definition and functional characterization of novel CAF subpopulations presents a significant challenge to the field and has been reviewed recently elsewhere [54,55].

#### 3.1.2. Desmoplasia and Clinical Outcomes

Many studies have demonstrated that desmoplasia in the TME is associated with poor patient outcomes. For example, increased stromal content (defined via semiquantitative assessment of stromal percentage in hematoxylin and eosin [H&E]-stained tumor sections) was associated with reduced relapse-free and overall survival among breast cancer patients [56,57]. Additionally, in orthotopic syngeneic murine models of PDAC, tumor desmoplasia was associated with reduced tumor perfusion and drug delivery, particularly in the setting of obesity [58]. These pre-clinical findings were validated in human patient samples, wherein obesity was associated with increased ECM deposition in the PDAC TME (by collagen I immunostaining) and poorer responses to adjuvant chemotherapy [58].

Other studies, however, indicate that the relationship between desmoplasia and cancer patient survival may be more complex. For instance, quantification of total tumor collagen content with Masson’s trichrome stain revealed that prostate cancer patients with either low- or high-stromal grade tumors experienced reduced relapse-free survival relative to patients with intermediate-stromal grade tumors [59]. Moreover, in PDAC, highly dense stroma (based upon subjective H&E staining patterns) [60] and a high “activated stromal cell index” (the ratio of αSMA expression to total collagen content) [61] were both positive prognostic indicators of patient survival. These discrepant results likely reflect the diverse methods by which tumor stroma can be quantified, as well as the heterogeneous and context-specific nature of desmoplastic stroma composition. They also foreshadow the variable effects of collagen-targeting approaches on tumor progression (see Section 4, below).

### 3.2. Contextual Functions of Individual Collagen Species in the TME

Although studies of desmoplasia en masse have yielded important insights into tumor initiation and progression, patient prognosis, and therapeutic responses, the incredible diversity of the collagen family indicates that a systematic investigation of individual collagen species in these processes is also warranted. Notably, a growing body of literature indicates that specific collagen molecules can act as double-edged swords in cancer-associated processes, eliciting pro-neoplastic effects in some settings and host-protective effects in others. Below, we highlight the context-specific roles of select collagen molecules in the TME, focusing particularly on those for which functional studies in highly desmoplastic cancer types (breast and prostate carcinomas, PDAC, and STS) were available.

#### 3.2.1. Collagen I

In cell culture models, collagen I promotes multiple aspects of desmoplastic tumor progression including cancer cell proliferation, survival, migration/invasion, and chemoresistance. For example, PDAC and prostate cancer cells cultured on collagen I coatings displayed increased proliferation and reduced apoptosis compared to cells cultured on other substrates such as fibronectin and tissue culture plastic [62,63]. Prostate, pancreatic, and mammary carcinoma cells grown on collagen I matrices also demonstrated hallmarks of the epithelial-mesenchymal transition (EMT), including e-cadherin suppression; upregulation of n-cadherin and the master EMT regulators SNAIL, SLUG, and ZEB1; and enhanced migratory capacity [64,65,66,67,68,69]. Furthermore, PDAC cells cultured in three-dimensional collagen I gels exhibited reduced sensitivity to gemcitabine, a PDAC standard-of-care agent, due to collagen I-induced upregulation of the chromatin modifier HMGA2 and ensuing changes in histone acetyltransferase expression [70,71].

In contrast, other studies have demonstrated that collagen I possesses anti-neoplastic functions in desmoplastic cancers. Wu et al. demonstrated that MDA-MB-231 invasive breast cancer cells exhibited cyclin E downregulation and delayed G1/S cell cycle progression when cultured in three-dimensional collagen I matrices but not on two-dimensional collagen I coatings [72]. Treatment of three-dimensional cell cultures with neutralizing antibodies to integrin β1, a collagen I receptor, rescued cyclin E expression and cell proliferation [72]. Protective effects of collagen I have also been documented in vivo where it impacts stromal cell populations such as CAFs and immune cells in addition to cancer cells per se. For example, using murine models of liver-metastatic pancreatic and colorectal cancer, Bhattarcharjee and colleagues demonstrated that collagen I produced by both lecithin-retinol acyltransferase (Lrat^+^) and αSMA^+^ hepatic stellate cell-derived CAFs (HSC-CAFs) physically impeded the growth of metastatic lesions [73]. However, HSC-CAFs also secreted hyaluronic acid and hepatocyte growth factor into the TME, thereby promoting tumor progression [73]. Taken together, these results indicate that soluble mediators originating from pro-tumorigenic CAFs in the TME can override the protective effects of collagen I in this context, and should be investigated for their suitability as therapeutic targets for metastatic desmoplastic cancers [73]. In a second study, Chen et al. used a series of elegant murine PDAC models to show that genetic deletion of *Col1a1* in αSMA^+^ myofibroblasts reduced intratumoral T cell content and suppressed T cell activation gene expression, potentially by promoting recruitment of myeloid-derived suppressor cells [74]. Although the observed T cell activation deficit was not confirmed flow cytometrically [74], these findings are consistent with a foundational report demonstrating that collagen I is a potent co-stimulator of effector CD8^+^ T cell expansion [75]. Interestingly, however, other studies have shown that collagen I can suppress T cell activation (and that of other leukocytes), predominantly by binding to the inhibitory receptor LAIR1 (leukocyte-associated immunoglobulin-like receptor 1) [76,77,78,79]. Thus, the effects of collagen I on immune cell recruitment, polarization, and function may be context-dependent, particularly in in vivo environments that may be replete with both stimulatory and inhibitory signals, and should be systematically explored in desmoplastic tumors.

#### 3.2.2. Collagen III

Functional studies of collagen III have predominantly been conducted in the setting of metastasis. For example, siRNA-mediated depletion of *COL3A1* in triple-negative breast cancer (TNBC) cells reduced cancer cell migration and invasion in vitro [80]. However, in vivo studies have reported an anti-metastatic role for collagen III. Specifically, using xenograft models of lung-metastatic head and neck squamous cell carcinoma (HNSCC), Di Martino et al. demonstrated that a pulmonary milieu rich in collagen III maintained disseminated cancer cells in a dormant state, preventing metastatic progression [81]. Mechanistically, the binding of extracellular collagen III to its receptor, cancer cell-expressed discoidin domain receptor I (DDR1), resulted in STAT1-mediated upregulation of *COL3A1*, thereby creating a positive feedback loop that sustained cellular dormancy [81]. Similar results were observed in mammary carcinoma models [81], suggesting that the tumor-suppressive effects of collagen III may be broadly applicable to lung-metastatic cancers. These findings were further substantiated by Brisson et al. who reported enhanced primary mammary tumor growth and pulmonary metastatic burden in *Col3a1* haploinsufficient mice compared to wild-type littermate controls [82]. Interestingly, maintenance of HNSCC cell dormancy was regulated by cancer cell-derived collagen III [81], whereas the anti-metastatic role of collagen III in breast cancer, although not definitively shown, was attributed to stromal fibroblasts [82]. Nevertheless, in both studies [81,82], collagen III deficiency remodeled the metastatic tumor ECM by promoting the deposition of “wavier”, less linear fibrillar collagen fibers. Taken together, these data clearly indicate that the cellular source of collagen molecules in the desmoplastic TME can have functional implications. Moreover, the apparent discrepancy between the role of collagen III in vitro [80] and in animal models [81,82] clearly indicates the need for in vivo investigation in this area.

#### 3.2.3. Collagen IV

Like other basement membrane collagens, proteolytic cleavage of collagen IV gives rise to bioactive peptide fragments called matrikines [1]. Interestingly, full-length collagen IV and its matrikines appear to play opposing roles in desmoplastic cancers, exhibiting pro- and anti-neoplastic effects, respectively. Specifically, breast cancer cells grown on tissue culture plates coated with full-length collagen IV displayed increased secretion of matrix metalloproteinase 9 (MMP9), a pro-metastatic protease [83]. However, the authors did not evaluate whether this phenotype also led to increased cancer cell migratory and/or invasive capacity [83]. Nevertheless, this report was substantiated by Fatherree et al. [84], who recently demonstrated that chemotherapy-mediated upregulation of collagen IV in the mammary carcinoma ECM enhanced cancer cell motility in a Src- and focal-adhesion kinase (FAK)-dependent manner [84]. Inhibition of Src/FAK signaling downstream of collagen IV attenuated chemotherapy-induced cancer cell migration, providing insights into mechanisms by which cytotoxic treatments may augment the metastatic potential of residual disease [84]. In contrast to the full-length molecule, collagen IV matrikines including arrestin, canstatin, and pentastatin (cleavage products of *Col4a1*, *Col4a2*, and *Col4a5*, respectively) inhibited tumor growth in xenograft models of prostate, mammary, and pancreatic carcinoma [85,86,87,88]. Mechanistically, these peptides exhibited anti-angiogenic effects such as induction of endothelial cell apoptosis, inhibition of endothelial tube formation, and/or reduction of tumor microvessel density in these contexts [85,86,87,88]. These encouraging findings led to the development of multiple gene therapy approaches seeking to deliver collagen IV matrikines to desmoplastic tumors in vivo, each of which successfully inhibited xenograft tumor growth in preclinical studies [89,90,91]. However, given the significant clinical challenges associated with currently available anti-angiogenic therapies such as bevacizumab (anti-vascular endothelial growth factor; VEGF), including induction of acute intratumoral hypoxia, impaired intratumoral drug delivery, and extremely modest improvements in overall survival [92,93,94,95,96], caution must be exercised if these novel approaches are to undergo further development for use in human patients.

## 4. More Than a Single Molecule: Heterogeneous Effects of Stromal-Targeting Approaches on Tumor Progression

Given the prevalence of collagen molecules in desmoplastic tumor stroma, reducing collagen deposition in the TME is considered a promising avenue for therapeutic intervention. However, these “stromal-targeting” approaches have had discordant effects on tumor progression depending on the cancer type, specific cellular and molecular target(s), and therapeutic modality in question. One possible explanation for these discrepancies—at least in part—is that these studies often refer to “collagen” as a single molecule, failing to account for the remarkable structural and functional diversity of the collagen superfamily, or the context-specific functions of individual species. Therefore, in the current section of this review, we discuss the efficacy of stromal-targeting strategies in desmoplastic tumors, focusing particularly on approaches that (1) deplete collagen-producing CAF populations in the TME, and (2) directly target collagen synthesis. Additional stroma-directed approaches are also under investigation, including those that block CAF activation and/or function (e.g., via inhibition of transforming growth factor β [TGFβ] signaling [97,98,99]), or target heterotypic interactions between cancer cells, CAFs, and/or other components of the TME (reviewed in [53]). However, these latter approaches generally impact tumor biology through more diverse mechanisms and are beyond the scope of this review. Still other approaches, such as administration of MMP inhibitors or bacterial collagenase, have been attempted with the goal of disrupting or normalizing collagen degradation/turnover. However, despite promising preclinical data, these strategies were unsuccessful in clinical trials due to toxicity (immunogenicity, off-target effects/non-substrate specificity, etc.) and poor efficacy. As such, we refer the reader to several excellent reviews for more detailed discussions of historical and future perspectives on this topic [100,101,102].

### 4.1. Reducing CAF Content in the Desmoplastic TME

As the primary cell type responsible for depositing the desmoplastic tumor ECM, at least in epithelial tumors, CAFs are a natural target for strategies that seek to reduce stromal content, and by extension, collagen deposition, in the TME. These approaches have shown considerable promise, particularly in pancreatic cancer. For example, conditional ablation of Fap^+^ stromal cells through systemic administration of diphtheria toxin (*Fap*^DTR/DTR^ mice) delayed tumor onset, impeded metastasis, and improved survival in the autochthonous KPC model of PDAC (*Kras*^LSL-G12D/+^*; Trp53*^LSL-R172H/+^*; Pdx1-Cre*) [103]. As tumors from *Fap*-deficient animals also exhibited increased necrotic cell death and elevated levels of lymphocyte infiltration, the authors speculated that Fap expression in the PDAC stroma may contribute to the poor efficacy of immunomodulatory therapies in some human patients [103]. Consistent with this observation, Feig and colleagues demonstrated that conditional depletion of Fap^+^ stromal cells enhanced the efficacy of immune checkpoint blockade (α-PD-L1 or α-CTLA-4) in the same animal model [104].

Successful stromal cell targeting has also been achieved with pharmacologic approaches. For instance, administration of the Hedgehog pathway inhibitor IPI-926 to PDAC-bearing mice depleted the desmoplastic tumor stroma and collagen I expression therein, thereby improving intra-tumoral drug delivery and reducing the incidence of metastasis [105]. In addition to small molecule inhibitors, docetaxel-conjugated nanoparticles that deplete αSMA^+^ myofibroblasts (or otherwise unspecified stromal cell populations), have also been developed; animal models of breast and pancreatic cancer have demonstrated that these approaches reduce tumor collagen I content, improve tumor perfusion, and reduce metastatic burden [106,107,108,109]. Importantly, these results are consistent with foundational studies describing inverse relationships between tumor collagen I fiber content and intratumoral macromolecular diffusion/perfusion [110,111,112]. One nanoparticle formulation, an albumin-bound paclitaxel particle (nab-paclitaxel/Abraxane), has also been evaluated in human clinical trials: when administered together with the PDAC standard-of-care agent gemcitabine, Abraxane elicited modest (~2-month) yet statistically significant increases in overall and progression-free survival compared to gemcitabine monotherapy [109,113,114]. Similar results were obtained in a phase III trial of women with metastatic breast cancer, wherein Abraxane treatment significantly prolonged progression-free survival compared to treatment with a standard paclitaxel formulation [115]. Taken together, these results support the notion that the desmoplastic stroma can impede drug delivery and promote immunosuppression, thereby representing potential avenues for improving the chemosensitivity of desmoplastic tumor types.

Paradoxically, however, other studies have shown a protective effect of fibroblasts in desmoplastic tumor stroma. For instance, despite reducing collagen I content, genetic depletion of αSMA^+^ myofibroblasts in the PKT murine model of PDAC (*Ptf1a*^Cre/+^; *Kras*^LSL-G12D/+^; *Tgfrb2*^Fl/Fl^) fostered the development of an immunosuppressive microenvironment characterized by an increased ratio of Foxp3^+^ T-regulatory cells to effector T cells [116]. In turn, αSMA-depleted PKT mice developed more invasive lesions and exhibited reduced survival; administration of α-CTLA-4 immunotherapy reversed these phenotypes and rescued effector T cell content [116]. Consistent with these findings, conditional genetic deletion of sonic hedgehog in the pancreatic epithelium significantly reduced αSMA^+^ myofibroblast content via a paracrine signaling mechanism, yet promoted the development of an undifferentiated, more aggressive PDAC tumor phenotype [117]. Interestingly, in contrast to an earlier study [105], administration of IPI-926 accelerated PDAC progression despite the use of an identical mouse model, dose, dosing schedule, and route of administration [117]. This surprising discrepancy was ultimately attributed to a longer treatment interval in ref. [117] (~4–5 months; chronic setting) compared to ref. [105] (<3 weeks; acute setting); as such, the authors speculated that there had been insufficient time to detect accelerated PDAC progression in the earlier study [117]. However, the IPI-926-treated mice also developed cachexia in the later study [117], potentially confounding the effects of Hedgehog inhibition on tumor biology and/or survival. Nevertheless, a phase II trial of IPI-926 for metastatic PDAC (ClinicalTrials.gov identifier: NCT01130142) was terminated early when an interim analysis demonstrated that the median survival time of patients receiving dual-agent IPI-926 + gemcitabine was shorter than that of patients treated with gemcitabine alone [118,119]. As Hedgehog inhibition likely exhibits pleiotropic effects in the setting of malignancy, it will be challenging to fully unravel the intricate relationship between this pathway and desmoplasia in PDAC [48]. Therefore, the future of Hedgehog pathway inhibitors in both PDAC and desmoplastic tumors more broadly remains uncertain.

### 4.2. Inhibition of Collagen Biogenesis

#### 4.2.1. The Collagen Synthesis Pathway

**Figure 1:**
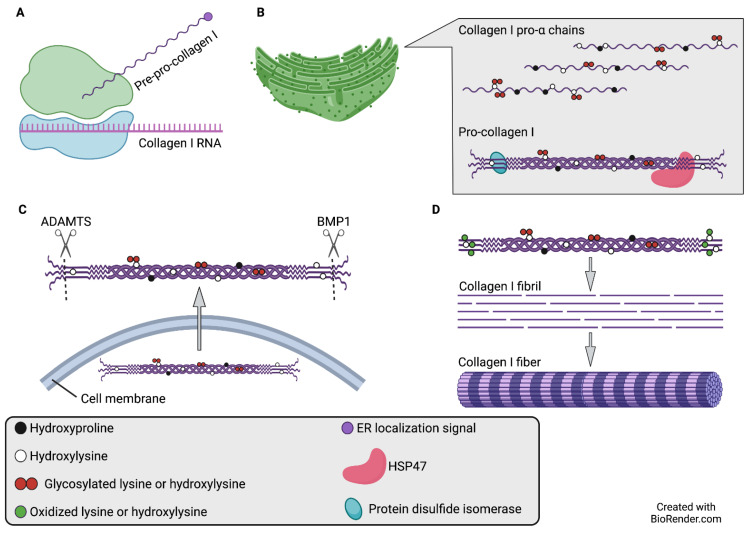
Synthesis of collagen I, a prototypical fibrillar collagen. (**A**) Collagen α chains are synthesized in ER-bound ribosomes as pre-pro-collagens. (**B**) Full-length pre-pro-collagen molecules are translocated to the ER and are known as pro-α chains when the ER localization signal is removed. Pro-α chains then undergo a series of post-translational modifications including proline hydroxylation (catalyzed by P4H), lysine hydroxylation (catalyzed by LH1–3), and/or lysine and hydroxylysine glycosylation (catalyzed by COLGALT1, COLGALT2, and/or LH1–3). Association of three post-translationally modified chains results in the formation of a pro-collagen molecule. Chaperone proteins such as HSP47 and protein disulfide isomerase facilitate pro-collagen stabilization. (**C**) Pro-collagen molecules are secreted into the extracellular space by exocytosis. (For simplicity, secretory granules are not shown.) Amino acid residues in pro-collagen N- and C- terminal regions, known as pro-peptides, are cleaved by ADAMTS and BMP1, respectively. Pro-peptide residues retained after cleavage are known as telo-peptides. (**D**) Individual collagen molecules self-assemble into collagen fibrils, which are stabilized by oxidation and crosslinking of telo-peptide lysines and hydroxylysines. This process results in a “quarter-staggered” fibril organization pattern. Multiple collagen fibrils aggregate to form collagen fibers, which are visible with light microscopy. Abbreviations: ADAMTS: a disintegrin and metalloproteinase with thrombospondin motifs; BMP1: bone morphogenetic protein 1; COLGALT: procollagen galactosyltransferase; ER: endoplasmic reticulum; HSP47: heat shock protein 47; LH: lysyl hydroxylase; P4H: prolyl-4-hydroxylase.

An alternative strategy by which to reduce collagen deposition in the TME is to directly inhibit collagen biogenesis. Collagen synthesis is a multi-step process that requires the activity of several post-translational modifying enzymes, many of which have been investigated as putative anti-neoplastic targets. Much of the available research concerning the collagen biogenesis pathway pertains specifically to fibrillar collagens, specifically collagen I (Figure 1).

Collagen α chains are synthesized in membrane-bound ribosomes and subsequently directed to the endoplasmic reticulum (ER) as pre-pro-collagens; these polypeptides are converted to pro-α chains following removal of the ER localization signal [120]. Within the ER, the nascent pro-α chains then undergo a number of post-translational modifications, including proline hydroxylation (catalyzed by collagen prolyl-4-hydroxylase), lysine hydroxylation (catalyzed by the lysyl hydroxylase [LH], also known as PLOD, family of enzymes), and lysine and hydroxylysine glycosylation (catalyzed by procollagen galactosyltransferases [COLGALT1 and COLGALT2] and glucosyltransferases [PLOD1-3 possess modest glucosyltransferase activity in addition to their lysyl hydroxylase activity; however, other as-yet unidentified glucosyltransferases may also be involved]) [37,120,121,122,123,124,125]. The association of three post-translationally modified pro-α chains results in the formation of a procollagen molecule, which is stabilized by collagen-binding chaperones such as heat shock protein 47 (*SERPINH1*) and protein disulfide isomerase [126,127,128]. Upon secretion into the extracellular space, the N- and C-pro-peptides of these procollagen molecules are proteolytically cleaved by ADAMTS (a disintegrin and metalloproteinase with thrombospondin motifs) family enzymes and bone morphogenetic protein-1 (BMP1), respectively [1]. The retained pro-peptide residues, also known as telo-peptides, constitute the non-collagenous domains of mature collagen molecules [129]. Subsequently, individual collagen molecules self-assemble into collagen fibrils, which can be visualized under electron microscopy [130]. Fibril stabilization, catalyzed by lysyl oxidase (LOX), is achieved by lysine and hydroxylysine crosslinking in the telo-peptides of adjacent, quarter-staggered collagen molecules [131,132]. Aggregation of multiple collagen fibrils results in the formation of collagen fibers, which are visible under light microscopy [130].

#### 4.2.2. Collagen Prolyl-4-Hydroxylase

Vertebrate collagen prolyl-4-hydroxylase (P4H) is an ER-resident α_2_β_2_ holoenzyme that modulates the proper three-dimensional folding of procollagen molecules. Three isoforms of the catalytic and peptide-binding α subunit have been described (P4HA1-3); P4HA1 is the predominant isoform in most human cells and tissues [133,134,135]. The P4H β subunit (P4HB) is identical to protein disulfide isomerase, a collagen chaperone [128]. Cytoplasmic P4Hs that hydroxylate HIF-1α, a master transcriptional regulator of cellular responses to hypoxia and well-established mediator of cancer progression, have also been identified (prolyl hydroxylase domain enzymes; PHD1/EGLN2, PHD2/EGLN1, and PHD3/EGLN3). However, with the exception of residues within the catalytic domain, there is no significant amino acid sequence homology between these enzymes and collagen P4Hs [136].

Prior work investigating the role of collagen P4H in desmoplastic cancers has primarily focused on isoforms 1 and 2 of the catalytically active α subunit. For example, knockdown of *P4HA1* and *P4HA2* in prostate [137] and breast [138] cancer, respectively, reduced cancer cell proliferation, invasion, and metastasis, both in vitro and in vivo. In breast cancer organoid cultures, *P4HA2* deficiency also reduced collagen I and IV deposition, but the authors did not investigate whether a similar effect also occurs in vivo [138]. Additionally, in PDAC, *P4HA1* knockdown significantly inhibited cancer cell proliferation, attenuated the expression of cancer stem cell markers, and increased the susceptibility of PDAC cell lines to gemcitabine [139]. P4HA1 deficiency also inhibited PDAC cell glycolysis, a well-established adaptive response to intratumoral hypoxia, potentially by downregulating the expression and activity of HIF-1α [139]. Consistent with these findings, Xiong and colleagues [140] recently reported that P4HA1 also promotes HIF-1α pathway activation in TNBC cells. Specifically, by reducing the levels of α-ketoglutarate and succinate, two metabolites that normally facilitate HIF-1α degradation under normoxic conditions, P4HA1 expression enhanced the stability of HIF-1α in TNBC stem cells [140]. Moreover, consistent with previously recognized relationships among tumor-initiating potential, chemoresistance, and metastatic colonization, *P4HA1* knockdown or P4HA inhibition increased the chemosensitivity of TNBC cells, both in vitro and in vivo, and reduced the incidence of metastases in docetaxel-treated mice [140]. Despite these encouraging results, however, the anti-neoplastic effects of P4HA cannot be extrapolated to all isoforms and tumor types. For instance, when expressed concomitantly with high levels of the serine protease *PRTN3*, low *P4HA2* levels predicted poor disease-free and overall survival in a PDAC patient cohort [141]. Moreover, upregulation of P4HA2 in melanoma and non-small-cell lung cancer models resulted in the extracellular release of collagen IV and XVIII fragments, ultimately suppressing angiogenesis and tumor growth in vivo [142]. Taken together, these data indicate that P4HA holds promise as a therapeutic target in a subset of desmoplastic cancers. However, a better understanding of the tumor type-specific functions of each P4HA isoform, particularly their effects on the expression and deposition patterns of individual collagen species, as well as the development of isoform-specific inhibitors, are important directions for future research.

#### 4.2.3. Lysyl Oxidase (LOX) and Lysyl Oxidase-like (LOXL) Proteins

The lysyl oxidase family catalyzes the covalent cross-linking of secreted collagen molecules, thereby conferring ECM stability and tensile strength [131,132]. LOX is secreted into the extracellular environment as a 50-kDa proenzyme (Pro-LOX) where it is proteolytically cleaved into a 32-kDa active form and an 18-kDa pro-peptide (LOX-PP) [143]. In vitro studies, primarily in breast cancer, have implicated catalytically active LOX in the promotion of cancer cell migration. Specifically, chromatin immunoprecipitation experiments demonstrated that LOX occupies the promoter of *SNAI2* (SLUG), a master regulator of EMT, where it upregulates *SNAI2* transcription [144]. Consistent with these results, treatment of invasive breast cancer cell lines with β-aminopropionitrile (βAPN), an irreversible inhibitor of LOX family enzymes, suppressed cell motility and adhesion [145]. Conversely, expression of either full-length LOX or the catalytically active peptide was sufficient to enhance the migratory and adhesive capacity of poorly invasive breast cancer cells [145]. In agreement with these cell-based findings, in vivo studies have demonstrated a role for LOX in the regulation of breast cancer progression and metastasis. For example, in a premalignant setting, surgically cleared murine (NOD/SCID) mammary fat pads were pre-conditioned with control or LOX-overexpressing fibroblasts and injected with human mammary epithelial cell organoids [146]. Compared to control fat pads, the stiffer, more highly cross-linked microenvironment of LOX-preconditioned fat pads more effectively promoted epithelial organoid growth and invasion [146]. Consistent with these data, inhibition of LOX activity with βAPN in an invasive breast cancer model (MMTV-Neu mice) reduced collagen cross-linking, fibrillar collagen deposition, and the formation of high-grade lesions [146]. Furthermore, in a metastatic setting, Pickup and colleagues demonstrated that highly metastatic PyMT^mgko^ tumors (in which the transforming growth factor β receptor *TGFBR2* is genetically depleted in mammary epithelial cells) displayed increased fibrillar collagen deposition, ECM stiffness, and LOX expression compared to poorly metastatic PyMT^fl/fl^ controls [147]. Administration of βAPN to tumor-bearing PyMT^mgko^ mice attenuated pulmonary metastatic burden and reduced the number of viable tumor cells in the systemic circulation, suggesting that LOX facilitates breast cancer metastasis by promoting cancer cell intravasation into the vasculature [147]. Finally, using an orthotopic 4T1-BALB/c model of bone-metastatic mammary carcinoma, Cox et al. showed that LOX stimulates the formation of osteolytic lesions, a major contributor to breast cancer patient morbidity and mortality, by enhancing both osteoblast differentiation and osteoclast activity [148]. Genetic depletion of LOX in the primary tumor (via shRNA-mediated LOX knockdown in implanted 4T1 cells), or systemic administration of LOX neutralizing antibodies, reversed osteolytic lesion formation and normalized osteoclast differentiation and function [148]. Taken together, these results illustrate that the role of catalytically active LOX in breast cancer metastasis is multifaceted and may depend on the specific cell type from which the enzyme is derived.

In addition to its role in metastasis, catalytically active LOX has also been implicated in the regulation of chemotherapeutic efficacy. Specifically, breast cancer, PDAC, and fibrosarcoma cells embedded in collagen I matrices demonstrated reduced sensitivity to doxorubicin or paclitaxel compared to the same cells cultured on plastic [149,150]. Consistent with these results, βAPN treatment or genetic LOX depletion enhanced the chemosensitivity of collagen I-embedded cells, potentially by improving rates of drug diffusion [149,150]. These findings were corroborated by in vivo experiments wherein pancreatic tumor-bearing mice treated with both gemcitabine and LOX neutralizing antibodies (αLOX) experienced significantly improved survival than animals treated with gemcitabine or αLOX alone [151]. Interestingly, tumors from mice treated with gemcitabine + αLOX exhibited a profound reduction in ECM protein expression and fibrillar collagen deposition, as well as a striking increase in tumor blood vessel dilation and immune cell infiltration (macrophages and neutrophils) [151]. Notably, however, these results were only obtained in the setting of early-stage disease, as the pro-survival effects of combination therapy on animals bearing late-stage pancreatic tumors were negligible [151]. Thus, it will be important for future pre-clinical and clinical studies of stromal-targeting approaches to discern the impact of these interventions in association with tumor stage at diagnosis. Moreover, given the inability of LOX inhibition to consistently suppress primary tumor growth [146,148,150,151,152], future research should center on the development of LOX-based combination therapies that control the progression of both primary and metastatic lesions. One strategy may be to combine βAPN with α-PD-1 immune checkpoint therapy; these agents synergistically suppressed primary tumor growth in murine PDAC models, likely in part by improving intratumoral T cell migration/infiltration [152]. Thus, the efficacy of combination LOX/immune checkpoint inhibition on PDAC metastasis, as well as on primary tumor growth in other desmoplastic tumor contexts, represents an important avenue for further study.

Unlike catalytically active LOX, LOX-PP exhibits tumor-suppressive functions in desmoplastic tumor contexts. In vitro, cancer cell-intrinsic LOX-PP expression inhibited the proliferation and invasion of prostate, pancreatic, and mammary carcinoma cells, and suppressed signaling pathways downstream of oncogenic Her2/Neu (breast cancer) and Ras (prostate and pancreatic cancer) [153,154,155]. LOX-PP also sensitized breast and pancreatic cancer cells to doxorubicin-mediated apoptosis [156] and reduced tumor growth in xenograft models [153,156,157]. Notably, this body of work [153,154,155,156,157] leveraged lentiviral expression systems to overexpress LOX-PP in cancer cells. Thus, it remains to be seen whether an effective LOX-PP delivery approach suitable for use in therapeutic applications can be developed.

Finally, in addition to LOX itself, the lysyl oxidase family is composed of the LOX homologs LOXL1-4. Most studies pertaining to the role of LOXL proteins in desmoplastic tumors have focused on LOXL2. Interestingly, LOXL2 facilitates cancer cell dedifferentiation, migration, and metastasis in a variety of desmoplastic cancer models (e.g., breast cancer, PDAC, muscle-derived rhabdomyosarcoma) [158,159,160,161,162,163] but exhibits diverse effects on ECM organization, even within specific cancer types. For instance, *Loxl2* genetic deletion in mammary epithelial cells had no impact on fibrillar collagen deposition or ECM biomechanical properties in the PyMT genetically engineered mouse model of breast cancer [160]. In contrast, systemic administration of LOXL2 neutralizing antibodies disrupted fibrillar collagen deposition and fiber linearity in established MDA-MB-231 breast cancer xenografts [159]. Together, these results suggest that the metastasis-promoting functions of LOXL2 may be independent of its ability to modulate ECM organization and stiffness, or that epithelial cell-derived LOXL2 is functionally distinct from LOXL2 expressed by other cell types. Despite these promising results, however, a recent study using orthotopic syngeneic models of PDAC reported that administration of LOXL2 neutralizing antibodies and ensuing reductions in fibrosis enhanced tumor progression through an unknown mechanism [164]. Thus, the future development of LOXL2-targeting strategies should be undertaken with caution.

#### 4.2.4. Lysyl Hydroxylases/PLODs

During collagen synthesis, the primary function of the PLOD family of enzymes (PLOD1-3) is to catalyze the hydroxylation of lysine residues on pro-collagen molecules. In normal tissues, hydroxylated lysines in collagen triple-helical domains serve as attachment sites for carbohydrate moieties, whereas those in telopeptide regions lead to the formation of hydroxylysine aldehyde-derived collagen crosslinks (HLCCs) that confer tissue stability and tensile strength [165,166]. Notably, all three PLOD family members catalyze hydroxylysine formation in collagen triple-helical domains; however, only PLOD2 has been shown to hydroxylate lysine residues in collagen telopeptides [124,165]. Furthermore, whereas PLOD-dependent lysine hydroxylation predominantly occurs in the ER [165,166], a recent study demonstrated that PLOD2 can also be secreted and catalyze hydroxylysine formation extracellularly [167].

Studies regarding the function of PLOD family enzymes in desmoplastic cancers have predominantly focused on the role of PLOD2. In TNBC, shRNA-mediated PLOD2 knockdown reduced TNBC cell migration and invasion in vitro, as well as primary tumor growth and metastasis in vivo [168]. Similar phenotypes were observed in autochthonous and allograft murine models of undifferentiated pleomorphic sarcoma, a commonly diagnosed and particularly lethal subtype of STS, and were dependent upon PLOD2 lysyl hydroxylase activity [49]. Mechanistically, PLOD2 genetic depletion or minoxidil-mediated PLOD2 inhibition resulted in the formation of more “mature” collagen networks, characterized by increased prevalence of higher molecular weight collagen I species (dimers and trimers) and the deposition of more linear fibrillar collagen fibers in the sarcoma ECM [49]. Thus, these promising studies in both epithelial and mesenchymal desmoplastic cancers indicate that the therapeutic potential of PLOD2 warrants further investigation in these contexts.

## 5. Conclusions and Future Directions

Despite the prevalence of desmoplasia in the TME of solid cancers, the functions of collagen molecules in this context are nuanced and diverse. As a result, a systematic investigation of the roles of individual collagen molecules in desmoplastic tumor biology is crucial. Future studies in this area should consider the cancer cell-intrinsic roles of specific collagen species, as well as the manner in which these molecules impact the dynamics and function of other cell types in the TME (e.g., endothelial cells; immune infiltrates). Additionally, we must consider the possibility that collagens and/or collagen-modifying enzymes may have different functions depending on the specific cell type from which they originate. Furthermore, although conventional cell culture experiments and xenograft models have provided a solid foundation for this field, future work must increasingly leverage more sophisticated approaches such as 3D organotypic cell culture systems and immunocompetent, genetically-engineered animal models. Finally, observations made in epithelial cancers need to be confirmed in mesenchymal tumors, which often exhibit distinct genetic/epigenetic landscapes and microenvironmental features from carcinomas.

With regard to further development and clinical assessment of “collagen-targeting” approaches (Table 2), future studies must comprehensively assess how these strategies impact the expression, deposition, organization, biomechanics, etc. of collagen molecules in the ECM, and, in turn, determine how these specific alterations more broadly impact tumor biology (including both cancer cell-intrinsic and-extrinsic effects, as well as cell–cell/cell–matrix interactions). They should also consider how these interventions alter the course of cancer progression when administered in acute (early-stage) vs. chronic (late-stage) disease settings. Together, these studies will facilitate the more precise identification of actionable interventions in patients with desmoplastic tumors, ultimately improving therapeutic responses and extending long-term survival.

## Figures and Tables

**Table 1:** Collagen species, sub-families, and normal tissue distribution.

Collagen Species	Sub-Family or Supramolecular Assembly Pattern ^1^	Distribution in Normal Mammalian Tissues	Reference(s)
Collagen I	Fibrillar collagen	Ubiquitous, but particularly in connective tissues, skin, the eye, and vasculature; healing wounds	[3,4]
Collagen II	Fibrillar collagen	Cartilage	[5]
Collagen III	Fibrillar collagen	Ubiquitous; supports cellular components of visceral organs and adipose tissue; healing wounds	[4,6,7]
Collagen IV	Network-forming collagen	Basal laminae	[8,9,10]
Collagen V	Fibrillar collagen	Cornea	[5]
Collagen VI	Beaded filaments	Ubiquitous, but particularly in skeletal muscle	[11]
Collagen VII	Anchoring fibrils	Anchoring filaments in basal laminae of stratified and complex epithelia	[12]
Collagen VIII	Network-forming collagen	Descemet’s membrane of the eye (basal lamina of corneal endothelium)	[5,13,14]
Collagen IX	FACIT ^2^	Cartilage	[15]
Collagen X	Network-forming collagen	Hypertrophic cartilage	[16]
Collagen XI	Fibrillar collagen	Cartilage	[5]
Collagen XII	FACIT	Cartilage; bone; dense connective tissue	[17]
Collagen XIII	Transmembrane collagen	Cutaneous tissue; neuromuscular junctions	[18,19,20]
Collagen XIV	FACIT	Virtually all collagen I-containing tissues (e.g., skeletal and cardiac muscle, dense connective tissue)	[17]
Collagen XV	Multiplexin	Ubiquitous; usually in basement membrane zones but occasionally in association with fibrillar collagens	[21,22]
Collagen XVI	FACIT	Cartilage; basement membrane zone of cutaneous tissue	[15,23]
Collagen XVII	Transmembrane collagen	Skin hemidesmosomes	[18,19]
Collagen XVIII	Multiplexin	Sub-epithelial basement membrane zones (e.g., kidney, placenta, lung, liver, skin)	[21,22]
Collagen XIX	FACIT	Interneurons	[24]
Collagen XX	FACIT	Embryonic structures (corneal epithelium, skin, cartilage, and tendon)	[25]
Collagen XXI	FACIT	Blood vessel walls of many highly vascularized fetal and adult tissues (e.g., brain, spinal cord, uterus)	[26]
Collagen XXII	FACIT	Tissue junctions (e.g., myotendinous junction in cardiac and skeletal muscle)	[27]
Collagen XXIII	Transmembrane collagen	Cutaneous tissue; excitatory neurons, especially of retina and olfactory bulb	[18,19,28]
Collagen XXIV	Fibrillar collagen	Differentiating osteoblasts; developing cornea	[29,30]
Collagen XXV	Transmembrane collagen	Neurons in brain regions associated with visual processing; hippocampus	[28]
Collagen XXVI	Unknown	Neonatal and adult testis and ovary	[31]
Collagen XXVII	Fibrillar collagen	Regions of endochondral ossification; cartilage; embryonic eye, coronary arteries, and dermis	[32,33,34,35]
Collagen XXVIII	Unknown	Non-myelinated regions of the peripheral nervous system	[36]

^1^ Collagen sub-family designations and supramolecular assembly patterns were obtained from refs. [1,37], except for those of collagens XXVI and XXVII, which were obtained from refs. [38,39]. ^2^ FACIT: Fibril-associated collagen with interrupted triple helices.

**Table 2:** Collagen-targeting approach clinical development pipeline.

Compound	Mechanism of Action	Cancer Type	Stage of Development	Effects on Tumor Progression
**CAF depletion approaches**				
IPI-926	Smoothened (Hedgehog pathway) inhibitor	PDAC	Preclinical (in vivo)	Improved drug delivery and reduced metastasis [105] Accelerated tumor progression [117]
		Advanced pancreatic adenocarcinoma	Phase Ib: NCT01383538 (IPI-926 + FOLFIRINOX ^1^)	Acceptable safety profile, but closed early due to IPI-926 toxicity in an independent phase II trial [118]
		Metastatic pancreatic cancer	Phase II: NCT01130142 (IPI-926 + Gemcitabine)	Closed early due to unacceptable safety profile of IPI-926 alone [118,119]
docetaxel-conjugated nanoparticles (e.g., nab-paclitaxel)	Prevents microtubule assembly; depletes αSMA^+^ myofibroblasts or other stromal cell population	PDAC; breast cancer	Preclinical	Improve tumor perfusion and reduce metastatic burden [106,107,108,109]
		Advanced PDAC	Phase I/II: NCT00398086 (nab-paclitaxel + Gemcitabine)	Tolerable adverse effects [109]
		Metastatic PDAC	Phase III: NCT00844649 (nab-paclitaxel + Gemcitabine)	Modest but significant survival increase vs. gemcitabine monotherapy [113,114]
		Metastatic breast cancer	Phase III	Significantly increased progression-free survival vs. standard paclitaxel [115]
**Collagen biosynthesis-targeting approaches**				
β-aminopropionitrile (βAPN)	Irreversible LOX-family enzymatic inhibitor	Invasive breast cancer	Preclinical (cell lines)	Decreased cell motility and adhesion [145]
		Breast cancer, PDAC, fibrosarcoma	Preclinical (cell lines)	Enhanced chemosensitivity [149,150]
		Breast cancer	Preclinical (in vivo)	Reduced high-grade lesion formation [146] Reduced pulmonary metastases and circulating tumor cells [147]
		PDAC	Preclinical (in vivo)	Suppressed primary tumor growth in combination with α-PD-1 ^2^ [152]
αLOX	LOX neutralizing antibody	Bone-metastatic mammary carcinoma	Preclinical (in vivo)	Reversed osteolytic lesion formation [148]
		PDAC	Preclinical (in vivo)	Improved survival (early stage disease); negligible effects (late-stage) [151]
LOX pro-peptide	Lentiviral overexpression	Breast cancer, PDAC	Preclinical (cell lines)	Improves chemosensitivity [156]
		Prostate, pancreatic, and mammary carcinoma	Preclinical (cell lines) Preclinical (in vivo)	Inhibits proliferation and oncogenic signaling [153,154,155] Reduces tumor growth [153,156,157]
αLOXL2	LOXL2 neutralizing antibody	Breast cancer	Preclinical (cell lines and in vivo)	Suppresses cell proliferation, adhesion, invasion, and migration; attenuates tumor growth [159]
		PDAC	Preclinical (in vivo)	Enhanced tumor progression [164]
Minoxidil	PLOD2 inhibitor	Fibrosarcoma, UPS ^3^	Preclinical (cell lines and in vivo)	Decreased cell migration and pulmonary metastasis [49]

^1^ FOLFIRINOX: 5-fluorouracil, leucovorin, irinotecan, oxaliplatin; ^2^ PD-1: programmed cell death protein 1; ^3^ UPS: undifferentiated pleomorphic sarcoma.
